# Effect of salpingectomy, ovarian cystectomy and unilateral salpingo-oopherectomy on ovarian reserve

**DOI:** 10.1007/s10397-016-0940-x

**Published:** 2016-03-28

**Authors:** Oybek Rustamov, Monica Krishnan, Stephen A Roberts, Cheryl T Fitzgerald

**Affiliations:** Department of Reproductive Medicine, St Mary’s Hospital, Manchester Academic Health Science Centre (MAHSC), Central Manchester University Hospital NHS Foundation Trust, Manchester, M13 0JH UK; Aberdeen Maternity Hospital, University of Aberdeen, Aberdeen, AB25 2ZN UK; Sheffield Teaching Hospitals, Royal Hallamshire Hospital, Sheffield, S10 2JF UK; Manchester Royal Infirmary, Central Manchester University Hospitals NHS Foundation Trust, Manchester, M13 9WL UK; Centre for Biostatistics, Institute of Population Health, Manchester Academic Health Science Centre (MAHSC), University of Manchester, Manchester, M13 9PL UK

**Keywords:** Salpingectomy, Ovarian cystectomy, Salpingo-oopherectomy, Ovarian reserve, AMH, AFC, FSH

## Abstract

Pelvic surgery can affect ovarian reserve, but estimates of the potential effect of different surgical procedures are lacking. This study examines the markers of ovarian reserve after different procedures in order to help the provision of informed consent before surgery. Anti-Müllerian hormone (AMH), antral follicle count (AFC) and follicle-stimulating hormone (FSH) of women with a history of salpingectomy, ovarian cystectomy or unilateral salpingo-oophorectomy were compared to those without history of surgery using cross-sectional data adjusting for patient and clinical factors in multivariable regression model. There were 138 women who had had salpingectomy, 36 unilateral salpingo-oopherectomy, 41 cystectomy for ovarian cysts that are other than endometrioma and 40 women had had excision of endometrioma. There was no significant difference in AMH (9 %; *p* = 0.33), AFC (−2 %; *p* = 0.59) or FSH (−14 %; *p* = 0.21) in women with a history of salpingectomy compared to women without surgery. Women with a history of unilateral salpingo-oophorectomy were found to have significantly lower AMH (−54 %; *p* = 0.001). These women also had lower AFC (−28 %; *p* = 0.34) and higher FSH (14 %; *p* = 0.06), the effect of which did not reach statistical significance. The study did not find any significant associations between a history of cystectomy, for disease other than endometrioma and AMH (7 %; *p* = 0.62), AFC (13 %; *p* = 0.18) or FSH. (11 %; *p* = 0.16). Women with a history of cystectomy for ovarian endometrioma had 66 % lower AMH (*p* = 0.002). Surgery for endometrioma did not significantly affect AFC (14 %; *p* = 0.22) or FSH (10 %; *p* = 0.28). Salpingo-oopherectomy and cystectomy for endometrioma cause a significant reduction in AMH levels. Neither salpingectomy nor cystectomy for cysts other than endometrioma has appreciable effects on ovarian reserve.

**Electronic supplementary material:**

The online version of this article (doi:10.1007/s10397-016-0940-x) contains supplementary material, which is available to authorized users.

## Introduction

Human ovarian reserve is determined by the size of oocyte pool at birth and an age-related decline in oocyte numbers thereafter. Both of these processes are largely under the influence of genetic factors, and to date, no effective interventions are available to improve physiological ovarian reserve [1]. However, various other environmental, pathological and iatrogenic factors appear to play a role, and consequently, it may be influenced either directly or indirectly. The use of chemotherapeutic agents, certain radio-therapeutic modalities and surgical interventions that damage the ovarian parenchyma can cause substantial damage to ovarian reserve [2, 3]. Estimation of the effect of each of these interventions is of importance in identifying lesser ootoxic treatment modalities.

Age is the main determinant of the number of non-growing follicles, accounting for 84 % of its variation. [4]. However, biomarkers that allow direct assessment of dynamics of growing follicles, anti-Müllerian hormone (AMH) and antral follicle count (AFC) may provide more accurate estimation of ovarian reserve [5]. Although these markers only reflect folliculogenesis of already recruited growing follicles, there appears to be a good correlation between their measurements and histologically determined total ovarian reserve [4]. Thus, the biomarkers can be utilised for the estimation of the effect of the above adverse factors on the primordial oocyte pool.

Surgical interventions that lead to disruption of the blood supply to the ovaries or involve direct damage to ovarian tissue may be expected to lead to a reduction in the primordial follicle pool. Indeed, a number of studies have reported an association between surgical interventions to the ovaries and a reduction in ovarian reserve [3]. However, given that both the underlying disease and surgery may affect ovarian reserve, disentanglement of the individual effects of these factors may be challenging and requires careful analysis. Here, we present a study that, in as far as is possible in cross-sectional data, intended to estimate the effect of tubal and ovarian surgery on ovarian reserve independently of underlying disease.

## Methods

The effect of salpingectomy, ovarian cystectomy and unilateral salpingo-oopherectomy on ovarian reserve was studied using serum biomarkers AMH, AFC and follicle-stimulating hormone (FSH) in a large cross-sectional study of patients referred for infertility management.

### Population

All women between ages of 20 to 45 who were referred to the Women’s Outpatient Department and the Reproductive Medicine Department of Central Manchester University Hospitals NHS Foundation Trust for management of infertility between 1 September 2008 and 16 November 2010 and had AMH measurement using the DSL assay ((DSL, Active MIS/AMH ELISA; Diagnostic Systems Laboratories, Webster, TX) were included. We excluded patients referred for fertility preservation and those with a diagnosis of polycystic ovaries (PCO) on transvaginal ultrasound scan which was defined as volume of one or both ovaries more than 10 ml. Patients with haemolysed AMH and/or FSH samples were not included in the analysis of these markers.

### Measurement of AMH

Blood samples for AMH were taken without regard to the day of women’s menstrual cycle. Serum samples were separated within 2 h of venipuncture in the Biochemistry Laboratory of our hospital and frozen at −20 °C until analysed in batches using the enzymatically amplified two-site immunoassay (DSL, Active MIS/AMH ELISA; Diagnostic Systems Laboratories, Webster, TX). All samples were processed strictly according to the manufacturer’s recommendations. The working range of the assay was up to 100 pmol/L and a minimum detection limit was 0.63 pmol/L. The intra-assay coefficient of variation (CV) (*n* = 16) was 3.9 % (at 10 pmol/l) and 2.9 % (at 56 pmol/l). The inter-assay CV (*n* = 60) was 4.7 % (at 10 pmol/l) and 4.9 % (at 56 pmol/l).

### Measurement of FSH

Women had measurement of basal FSH, luteinizing hormone (LH) and oestradiol levels (E2) during early follicular phase (days 2–5) of their menstrual cycle as part of their initial workup. Blood samples were transported to the Biochemistry Laboratory within 2 h of venipuncture for sample processing and analysis. Specific immunoassay kits (Cobas, Roche Diagnostics, Mannheim, Germany) and an autoanalyser platform were used (Roche Modular Analytics E170, Roche, USA) for analysis of FSH. The intra-assay CV was 6.0 % and inter-assay CV was 6.8 %.

### Measurement of AFC

Measurement of AFC was conducted in patients referred for assisted conception. The department used a stringent methodology for the assessment of AFC, which consists of counting all antral follicles measuring 2–6 mm in longitudinal and transverse cross sections of both ovaries using transvaginal ultrasound scanning (Toshiba Nemio F2534312) at early follicular phase of the menstrual cycle. The AFC with the closest date to AMH measurement was selected. The ultrasound assessments were conducted by a number of qualified sonographers, who used the same methodology for the measurement of AFC.

### Definitions and groups

Women’s body mass index (BMI) was categorised using standard NHS reference ranges: Underweight (<18.5), Normal (18.5–24.9), Overweight (25–29.9) and Obese (30–40) [8]). The causes of infertility were established by searching the referral letters, clinical notes and letters generated following clinic consultations. Women with a history of bilateral tubal block, which was confirmed by laparoscopic dye test, and patients with a history of bilateral salpingectomy were categorised as having severe tubal factor infertility. Women with unilateral tubal patency or unilateral salpingectomy were categorised as having mild tubal factor infertility. Severe male factor infertility was defined as azoospermia or severe oligospermia (<1 mln sperm sample) and partners with abnormal sperm count that do not meet the above criteria being classified as having mild male factor infertility.

Patients with reproductive surgery were categorised as having a history of salpingectomy, unilateral salpingo-oopherectomy, cystectomy for ovarian cysts other than ovarian endometrioma and cystectomy for endometrioma. In our department, stripping of cyst wall with subsequent diathermy of bleeding areas of the cyst bed is the standard method for excision of endometriotic cyst. However, the dataset did not contain data on surgical techniques and, therefore, we were not able to investigate the effect of specific surgical procedures.

### Statistical analysis

A multivariable regression model that included age, ethnicity, endometriosis, presence of ovarian endometrioma, causes of infertility, and tubal and ovarian surgery was fitted to the logarithm of each of the ovarian reserve markers: AMH, AFC and FSH. The age on the day of the measurement of each of the marker of ovarian reserve (AMH, AFC and FSH) was included in the model as a quadratic function following centering to 30 years of age. Preliminary analysis of AMH, AFC and FSH indicated that logarithmically transformed values with a quadratic age term provided adequate fits. Differences between the groups were considered significant at *p* < 0.05. Interactions between all explanatory variables were tested at a significance level of 0.01.

## Results

In total, 3179 women were included in the study. The AMH measurements of 66 women were excluded due to haemolysed samples or delay in processing the samples, leaving 3113 women for analysis. Of women, 1934 had AFC and 2580 had FSH. The mean (±SD) age, AMH, AFC and FSH of patients were 32.8 ± 4.5, 17.3 ± 14.8, 13.9 ± 6.2 and 8.0 ± 7.5, respectively. There were 138 women who had unilateral or bilateral salpingectomy, 36 women with a history of unilateral salpingo-oopherectomy, 41 women with a history of cystectomy for ovarian cysts other than endometrioma and 40 women had cystectomy for endometrioma. The results of the regression analysis on the effect of reproductive surgery on AMH, AFC and FSH are shown in Table 1.

**Table 1:** Multivariable regression analysis

	Number	Coef	95 % CI	*p*
Salpingectomy				
AMH	2128	0.094	−0.097, 0.285	0.333
AFC	1697	−0.027	−0.126, 0.072	0.595
FSH	1929	−0.056	−0.143, 0.032	0.210
Oopherectomy				
AMH	3049	−0.540	−0.868, −0.213	**0.001**
AFC	1946	−0.280	−0.857, 0.298	0.342
FSH	2546	0.139	−0.006, 0.284	0.060
Cystectomy other				
AMH	2128	0.075	−0.226, 0.376	0.626
AFC	1697	0.130	−0.064, 0.323	0.189
FSH	1929	0.110	−0.044, 0.265	0.161
Cystectomy endometrioma				
AMH	2128	−0.667	−1.081, −0.252	**0.002**
AFC	1697	0.144	−0.089, 0.376	0.225
FSH	1929	0.103	−0.084, 0.290	0.281

The fitted coefficient (log difference between the group indicated and all other patients), 95 % confidence interval and associated *p* value adjusted for age, ethnicity causes of infertility, endometriosis (without endometrioma) and endometrioma

Statistically significant values (*p*<0.005) are provided in bold

The analysis did not find any significant differences in AMH (increase of 9 %; *p* = 0.33), AFC (−2 %; *p* = 0.59) and FSH (−14 %; *p* = 0.21) between women with a history of salpingectomy and those without surgery (Table 1). Women with a history of unilateral salpingo-oopherectomy were found to have significantly lower AMH (−54 %; *p* = 0.001) and AFC (−28 %; *p* = 0.34) and increased FSH (14 %; *p* = 0.06), and the effect on AMH reached statistical significance (Table 1). The study did not find a significant association between previous history of ovarian cystectomy that was for disease other than endometrioma and AMH (7 %; *p* = 0.62), AFC (13 %; *p* = 0.18) or FSH (11 %; *p* = 0.16) (Table 1). Women with a history of ovarian cystectomy for endometrioma had 66 % lower AMH (*p* = 0.002) levels but the effects on AFC (14 %; *p* = 0.22) and FSH (10 %; *p* = 0.28) were not significant (Table 1).

## Discussion

In salpingectomy, tubal and ovarian branches of uterine arteries are often excised alongside the mesosalpynx and, hence, it is believed that disruption to blood supply to ovaries may lead to reduction of ovarian reserve. However, in our study, we did not observe an appreciable association between salpingectomy and any of the biomarkers of ovarian reserve suggesting this surgery does not affect ovarian reserve. These findings are supported by a longitudinal study that assessed the effect of tubal dissection to AMH, AFC and FSH (*n* = 49) [6]. There were no differences between preoperative and 3-month postoperative measurements with median AMH (1.5 vs. 1.4; *p* = 0.07), AFC (8.4 ± 3.7 vs. 7.9 ± 4.1; *p* = 0.09), FSH (7.6 ± 2.1 vs. 7.7 ± 2.1; *p* = 0.10). Silva et al. assessed the effect of tubal ligation (*n* = 52) in longer term postoperative period (1 year) and reported that median AMH (1.43, IQR 0.63–2.62 vs. 1.30, IQR 0.53–2.85; *p* = 0.23) and mean AFC (8, IQR 5–14 vs. 11, IQR 7–15; *p* = 0.12) did not change significantly [7]. Thus, our results along with other published evidence suggest that salpingectomy or tubal division does not have an adverse effect on ovarian reserve. Therefore, advising salpingectomy for various indications, including treatment of tubal pathology, sterilisation or opportunistic procedure as part of risk reduction strategy in ovarian carcinoma appears to be safe with regard to preserving ovarian reserve.

Although salpingo-oopherectomy is rare in women of reproductive age, significant ovarian pathologies and acute diseases such as ovarian torsion may necessitate unilateral salpingo-oopherectomy. There is plausible causative relationship between this surgery and ovarian reserve, although to our knowledge there is no previous published evidence. We found that women with history of unilateral salpingo-oopherectomy have significantly lower AMH (−54 %) suggesting the surgery has considerable negative impact on ovarian reserve measured with this biomarker. Similarly, the patients with a history of salpingo-oopherectomy had considerably higher FSH (13 %) and lower (−24 %) AFC. However, these did not reach statistical significance which may be due to small sample size and relative poor discriminatory power of AFC and FSH compared to that of AMH. The important clinical question in the management of patients with salpingo-oopherectomy is whether these patients have comparable reproductive lifespan or experience accelerated loss of oocytes resulting in premature loss of fertility, as this would allow appropriate preoperative counseling of patients regarding the long-term effect of the surgery on fertility and age at menopause. There is a need for studies with a larger number of patients, preferably using long-term longitudinal data, to investigate this question.

In women with a history of ovarian cystectomy for cysts other than those due to endometrioma, we did not observe any significant association between surgery and markers of ovarian reserve. However, women that had ovarian cystectomy for endometrioma appear to have significantly lower AMH (−66 %) compared to those without a history of surgery.

During the last few years, a number of studies have assessed the effect of excision of endometrioma on AMH [8–10]. The studies have been summarised by a recent systematic review, which concluded that excision of endometrioma results in damage to ovarian reserve [3]. Further studies evaluated the mechanism of damage, and these suggest that coagulation for the purpose of hemostasis as well as stripping of the cyst wall may cause direct damage to ovarian reserve. Sonmezer et al. compared the effect of diathermy coagulation (*n* = 15) for hemostasis compared to the use of hemostatic matrix (*n* = 13) in a randomised controlled trial and reported that the use of diathermy coagulation is associated with significantly lower AMH measurements (1.64 ± 0.93 vs. 2.72 ± 1.49 ng/mL) in the first postoperative month [11].

Similarly, stripping of the cyst wall also appears to have a detrimental effect on ovarian reserve due to inadvertent removal of ovarian tissue [12]. Using histological data, Roman et al. demonstrated that normal ovarian tissue was removed in 97 % specimens of surgically removed endometriomata [13]. Furthermore, it appears that ovarian cortex containing endometrioma appears to have significantly reduced density compared to normal ovarian cortex, and therefore, loss of oocyte containing normal ovarian cortex may be unavoidable in cystectomy for endometrioma [14]. Matsuzaki et al. conducted a histological assessment of cystectomy specimens and found that normal ovarian tissue adjacent to cyst wall was found in 58 % (71/121) of patients with endometrioma, whereas normal ovarian tissue was excised in 5.4 % (3/56) following cystectomy for other benign cysts [15]. Donnez et al. reported the use of combined stripping and vaporization technique was safe with regard to protecting an ovarian reserve [16]. More recently, Ata et al. reported that mean decline of AMH levels was less in suturing and haemostatic sealent technique compared to bipolar desiccation suggesting energy sources may have more detrimental effect on ovarian reserve [17].

Interestingly, contrary to AMH levels, the surgery does not seem to affect AFC measurements. A recent systematic review of 13 studies reported that AFC did not change following excision of endometrioma compared to that of prior surgery [18]. Similarly, our data did not show a significant difference in AFC measurements in patients with a history of excision of endometrioma, whilst AMH measurements of the patients with surgery was significantly (66 %) lower. This suggests that either (a) there is increased expression of AMH in the presence of endometrioma and hence the dramatic decline following cystectomy or (b) the performance characteristics of AFC is not sufficiently precise for the detection of change between the measurements. We believe exploration of these questions further may improve our understanding of the pathophysiology of ovarian endometriosis and performance of the markers of ovarian reserve in the presence of the disease.

In summary, in our study, women with a history of cystectomy for endometrioma had significantly lower AMH, whilst those had cystectomy for other benign cysts do not appear to have lower AMH. In view of our findings and other published research evidence, it seems clear that cystectomy for endometrioma results in a significant reduction in AMH levels.

### Strengths and limitations

The published studies have used longitudinal data comparing biomarkers before and after cystectomy and provide reliable estimates on the effect of the intervention on ovarian reserve. However, data on the effect of salpingectomy and unilateral salpingo-oopherectomy is lacking. In addition to a reevaluation of the effect of cystectomy, this study has assessed the impact of salpingectomy and unilateral salpingo-oopherectomy on markers of ovarian reserve. In contrast to published studies, this study employed analysis of cross-sectional data. Although we have adjusted for all the measured confounders, we cannot be certain that all relevant factors have been included and the apparent effects of surgery here may be causally related to some unmeasured factor related to the decision whether or not to intervene surgically. In patients with a history of cystectomy for endometrioma, we estimated independent effects of pathology and surgery providing important data for preoperative counseling.

It is important to note that the study evaluated the effect of surgery using retrospective data which has limitations due variation in recording of surgical history and missing data. Recent studies showed that AMH measurements may be prone to an inaccuracy due to methodological issues [19, 20]. However, this appears to be largely confined to initial Gen II AMH Assay [20, 21]. The study employed the data obtained using first-generation DSL AMH assay, which appears to provide more reproducible measurements [20, 22].

**Fig. 1:**
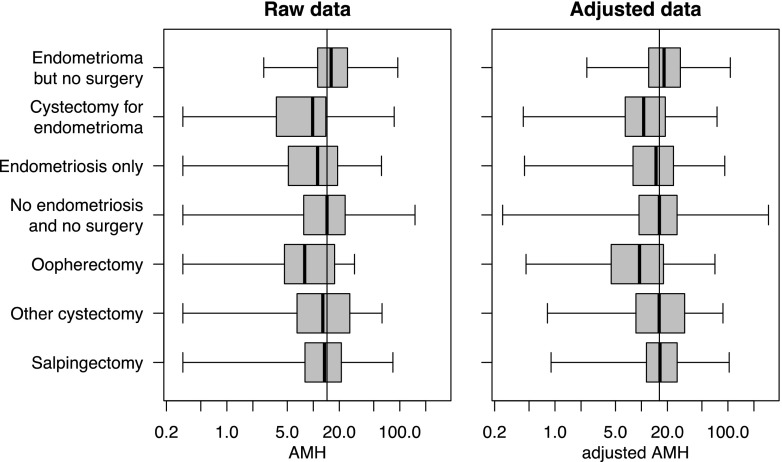
AMH by treatment groups. *Left hand panel* shows the raw data AMH measurement (in pmol/L) and the right hand panel the AMH adjusted for age, ethnicity, causes of infertility, endometriosis, endometrioma and surgery using the multivariable regression model for the various treatment groups

It is important to note although the effects are significant in a population level, there is considerable variation between individuals in the effects of surgery (Fig. 1). It is not clear whether this variability represents measurement error arising from the assays and sampling procedures, or true inter-individual differences in the effects between women. Thus, clinicians should exercise caution in predicting the effect of surgery on the ovarian reserve of individual patients.

## Conclusion

This multivariable regression analysis of retrospectively collected cross-sectional data suggests that neither salpingectomy nor ovarian cystectomy for cysts other than endometrioma has an appreciable effect on ovarian reserve determined by AMH, AFC and FSH. In contrast, salpingo-oopherectomy and ovarian cystectomy for endometrioma appear to have a significant detrimental impact on ovarian reserve. On the basis of findings of this study and other published studies, women undergoing reproductive surgery should be counseled with regard to the potential adverse effect of the surgery on their ovarian reserve.

## Electronic supplementary material

ESM 1 (DOC 112 kb)
